# Raster-scanned carbon ion therapy for malignant salivary gland tumors: acute toxicity and initial treatment response

**DOI:** 10.1186/1748-717X-6-149

**Published:** 2011-11-02

**Authors:** Alexandra D Jensen, Anna V Nikoghosyan, Swantje Ecker, Malte Ellerbrock, Jürgen Debus, Klaus K Herfarth, Marc W Münter

**Affiliations:** 1 Dept. Radiation Oncology, University of Heidelberg, INF 400, 69120 Heidelberg, Germany; 2 Dept. of Medical Physics, Heidelberg Ion Beam Therapy Centre (HIT), INF 450, 69120 Heidelberg, Germany

## Abstract

**Background and purpose:**

To investigate toxicity and efficacy in high-risk malignant salivary gland tumors (MSGT) of the head and neck. Local control in R2-resected adenoid cystic carcinoma was already improved with a combination of IMRT and carbon ion boost at only mild side-effects, hence this treatment was also offered to patients with MSGT and microscopic residual disease (R1) or perineural spread (Pn+).

**Methods:**

From November 2009, all patients with MSGT treated with carbon ion therapy were evaluated. Acute side effects were scored according to CTCAE v.4.03. Tumor response was assessed according to RECIST where applicable.

**Results:**

103 patients were treated from 11/2009 to 03/2011, median follow-up is 6 months. 60 pts received treatment following R2 resections or as definitive radiation, 43 patients received adjuvant radiation for R1 and/or Pn+. 16 patients received carbon ion treatment for re-irradiation. Median total dose was 73.2 GyE (23.9 GyE carbon ions + 49,9 Gy IMRT) for primary treatment and 44.9 GyE carbon ions for re-irradiation. All treatments were completed as planned and generally well tolerated with no > CTC°III toxicity. Rates of CTC°III toxicity (mucositis and dysphagia) were 8.7% with side-effects almost completely resolved at first follow-up.

47 patients showed good treatment responses (CR/PR) according to RECIST.

**Conclusion:**

Acute toxicity remains low in IMRT with carbon ion boost also in R1-resected patients and patients undergoing re-irradiation. R2-resected patients showed high rates of treatment response, though follow-up is too short to assess long-term disease control.

## Introduction

Malignant salivary gland tumors (MSGT) are rare and account for about 3-5% of head and neck cancers. They include a heterogeneous group of various histological subtypes with high-grade tumors such as mucoepidermoid carcinoma and adenoid cystic carcinoma (ACC) [1]. MSGTs are generally characterized by a rather slow pattern of growth, perineural spread and high propensity for hematogenous metastases, therefore outcome is still hampered by the incidence of distant metastases. Standard therapy of localized high-grade MSGT consists of complete surgical resection and adjuvant radiation in high-risk situations (R+ or close margin, perineural spread, large tumors (T3/4), or nodal metastases) [2-4]. Radiation doses of > 60 Gy or even 66 Gy are recommended to achieve local control [5-7].

Local control in MSGT was significantly improved by high-precision radiotherapy techniques, dose-escalation and high-LET radiation [8-12].

Intensity-modulated radiation therapy (IMRT) as well as fractionated stereotactic RT could already improve local control as compared to conventional RT techniques achieving 3-year PFS rates of 38% even in large ACCs [13].

The highest local control rates at 75 - 100% [10,12] are achieved by neutron radiation albeit at the cost of significant late toxicity. Heavy ion therapy using carbon ions however, showed a mild toxicity profile, no CTC°III late toxicities and very few °III acute reactions were observed [11]. Proton radiotherapy yielded an overall local control of 93% at 5 years; however, the authors noted several °III as well as one °V late toxicity (temporal lobe necrosis) [14]. A retrospective analysis of patients treated with neutrons reported slightly disappointing local control rates of 57% at 5 years accompanied with significant late toxicity (14% > CTC °III) [10]. IMRT plus carbon ion boost for ACC showed very favorable results without the dreaded late toxicity resulting in local control rates of 78% at 4 years [9]. These results in turn led to the acceptance of this regimen as the standard treatment for ACC in Germany. A recent update of patients with ACC treated with this regimen between 1997 and 2008 confirmed initial results with consistently low treatment-related side effects [9,15,16].

Charged-particle therapy promises improved results for all types of malignant salivary gland cancers. We would like to report early toxicity in a patient cohort including various R1-resected and re-irradiated MSGT of the head and neck.

## Patients and methods

103 patients were treated with raster-scanned carbon ion therapy from November 2009 to March 2011. Toxicity was assessed at completion of treatment and on each follow-up visit. Treatment response was evaluated according to RECIST based on contrast-enhanced MRI-scans where applicable.

### Patients

Patients with histologically confirmed or surgically removed malignant tumors of the salivary glands (head and neck) were offered combined IMRT plus C12-boost in cases where tumors were either surgically inoperable or partially (R2) resected. In contrast to our initial experience [9], also patients with microscopic residues (R1) or perineural invasion (Pn+) were offered the combination regimen. Prior RT was not an exclusion criterion if another course of radiation therapy was justifiable.

### Radiotherapy

#### Immobilization/planning examinations

Patients were immobilized with scotch cast or thermoplastic head masks with shoulder fixation. Planning examinations consisted of CT and contrast-enhanced MRI for 3D image correlation.

#### Target volumes/dose prescription

##### Primary treatment

CTV1 (carbon ion boost) includes the macroscopic tumor/prior tumor bed, CTV 2 typical pathways of spread/ipsilateral nodal levels (II/III), further levels are included as indicated details of target volume definition are described in our current standard protocol [17].

We prescribed a dose of 24 GyE carbon ions to the CTV1 (coverage: 95% prescription isodose). CTV2 receives 50 Gy IMRT (coverage > 90% of prescription isodose).

##### Re-irradiation

Patients undergoing re-irradiation receive carbon ion therapy alone; CTV1 is limited to the visible tumor only. Doses are prescribed highly individually depending on prior RT and interval between the two treatments.

#### Treatment and patient position control

##### Carbon ions

Carbon ion therapy is given in active beam application in the raster-scanning method [18] at the Heidelberg Ion Beam Therapy Centre (HIT). Inverse treatment planning is carried out on a dedicated planning system (Siemens TPS^®^) including biological RT optimization tools. Intensity-modulated particle therapy (IMPT) and single-beam optimization (SBO) techniques are used for plan generation in MSGTs.

Treatment is given at 3 GyE per fraction (5 x/week), daily image guidance consists of orthogonal x-rays in treatment position. An automatic 2D-3D pre-match is carried out (Siemens syngo PT treatment) and verified and manually adjusted online by the radiotherapist based on bony anatomy. Shifts were always corrected using a robotic table in six degrees of freedom.

##### IMRT

IMRT is given in 25 fractions (5 x/week) after inverse planning with the optimization tool KonRad MRC^® ^(Siemens OCS) on a 6 MV linear accelerator in step-and-shoot technique or on a 6 MV tomotherapy unit.

Regular image guidance is carried out as MV cone-beam CT or MV portal images. Total doses include doses by daily image guidance with MV CT.

#### Treatment schedule/follow-up

Most patients received upfront carbon ion boost followed by IMRT corresponding to a total dose of approximately 74 GyE (total treatment duration: approximately 7 weeks).

First follow-up including fibreoptic examination and MRI is carried out 6 weeks post completion of RT. Further MRI controls follow 3, 6, and 12 months thereafter, in 6 monthly intervals until 2 years post RT, then in yearly intervals.

### Analysis

Evaluation of toxicity was carried out according to NCI CTCAE v.4.03, treatment was evaluated using the RECIST-criteria if applicable (R2-resected or inoperable tumors) [19].

## Results

Treatment has been completed in 103 patients. Median age in our cohort is 56 years, median follow-up 6 months. Diagnostic images of the first follow- are available in 90 patients. Two patients from abroad are lost to follow-up.

Sixty patients had a visible residual tumour or had not undergone prior surgery (group 1), 43 patients had undergone R+ resections and/or showed extensive perineural spread (Pn+) (group 2). Eight out of 43 patients (group 1) and 26/60 patients (group 2) were treated for local relapse following initial surgery. Eighty-seven patients received treatment as part of their primary therapy. Sixteen patients received carbon ion therapy as re-irradiation.

The majority of patients was treated for ACC and advanced tumor stages (table 1).

**Table 1:** patient baseline characteristics

	Re-irradiation	visible residual tumor	R1/Pn+ resected tumors
**patient number**	16	60	43
prior surgery	5	26	43
visible residual/no prior surgery	15	60	0
R1/Pn+	1	0	43
recurrent tumors	16	26	9
re-irradiation	16	15	1
			
**Stages **(TNM 7th edition 2010)			
T1		1	8
T2		2	7
T3	4	10	7
T4	12	45	15
Tx		1	6
Not applicable		1	
			
N0			
N1			1
N2a		2	
N2b	1	3	4
N2c		1	1
			
**histology**			
adenoid cystic carcinoma	13	52	38
mucoepidermoid carcinoma	1	2	3
acinic cell carcinoma	1	3	
adenocarcinoma		1	1
squamous cell carcinoma	1	1	1
MSGT NOS		1	
			
**site**			
base of skull	6	9	1
intracranial extension		2	
orbit	1	4	
orbit/pterygopalatine fossa	4	2	1
petrous bone		1	
nasal cavity		1	1
paranasal sinus	3	16	7
maxilla		1	2
palate		1	6
planum buccale			1
nasopharynx		3	
oropharynx		1	
external auditory canal	1	2	
parotid gland	1	12	17
submandibular gland		2	5
sublingual gland			2
base of tongue		2	
lacrimal gland		1	

All except 3 patients received radiation only, one patient with extensive ACC received cetuximab weekly in addition, two patients with squamous cell carcinoma cisplatin 40 mg/m^2 ^body surface weekly throughout therapy (1 patient as primary therapy, 1 patient for re-irradiation).

### Primary treatment

Median target volumes (n = 87 pts) were 368 ml (IMRT; CTV2) and 128.6 ml (carbon ion boost; CTV1), median total dose was 73.2 Gy. Ten patients with tumors close to or crossing midline received bilateral cervical irradiation, for three patients with positive cervical lymph nodes and perinodal infiltration, our standard dose prescription was changed to 54 - 56 Gy to the CTV2 and 18 GyE C12.

Seventeen carbon ion plans needed to be optimized as IMPT, carbon ion treatment was mostly applied over 2 non-coplanar beams (table 2).

**Table 2:** treatment characteristics

	re-irradiation	range	primary irradiation	range
**patient number**	16		87	
				
**median dose (GyE/Gy)**				
C12	44.7		23.9	17.4 - 24.4
IMRT	48.8		49.3	47 - 56.3
total dose	44.9	36.2 - 72.7	73.2	69.9 - 75.3
				
**C12**				
IMPT	5		17	
SBO	10		70	
# of fractions	15	8 - 20	8	6 - 8
				
**IMRT**				
step& shoot IMRT	1			81
Tomotherapy	0			6
				
**treatment volumes (ml)**				
CTV1	55.2	9.2 - 178.7	128.6	32.6 - 468.6
CTV2	221.2	108.6 - 333.8	368.0	100.2 - 1246.8
				
**prior RT**				
prior C12-irradiation	6 pts			
median prior dose (GyE/Gy)	70	50 - 72		
median re-RT dose (GyE/Gy)	44.9	36.2 - 72.7		
median cumulative dose (GyE/Gy)	113	59 - 133		
time interval (months)	66	16 - 266		

### Re-irradiation

Sixteen patients received carbon ion therapy for re-irradiation of recurrent disease at the former field edge (2 pts), within the dose gradient to the optic nerve (2 pts), and in-field (12 pts). Median interval between the two RT courses was 66 months, median prior radiation dose 70 Gy. Six patients had initially received 72 GyE as combined treatment with carbon ion boost. Median re-treatment dose was 44.9 GyE (table 2).

One patient had initially received electrons to a small parotid field > 20 years ago, therefore this patient also received the standard IMRT + C12-boost concept despite prior RT (table 2). Dose prescription for re-irradiation was highly individual prior dose and respecting the patients' preferences after detailed discussion of risks and benefits.

#### Treatment tolerance/toxicity

Treatment was well tolerated despite comparatively long treatment times per session (set-up, position verification, treatment for carbon ions approx. 45 min). There was no treatment break in any patient except one, a lady with Miur-Torre syndrome showing pronounced swelling of the irradiated area (floor of mouth/pharynx) necessitating elective tracheal tube insertion and treatment interruption for 3 fractions.

Treatment-related acute effects as assessed at completion of the radiation therapy course were generally mild with mucositis CTC °III occurring in 9/103 pts (8.7%); despite extensive treatment fields, no > grade I xerostomia was found. Seventy-two patients (69.9%) developed changes up to complete loss of taste gradually resolving until the first follow-up. Twenty-one patients (20.4%) developed middle ear effusions/otitis requiring tympanostomy, 11 pts (10.7%) otitis externa. Seventeen patients had a facial nerve palsy due to extensive surgical procedures or tumor compression, 3 patients showed improvement of cranial nerve palsies (palsy of the IIIrd, Vth, VIth cranial nerv) during the course of radiotherapy completely resolving at first follow-up in 2 patients. We observed radiation-induced erythema in 86 patients, (62 pts °II, 25 pts °I), one of them CTC°III, small dry epitheliolyses were seen in 20 patients (19.4%) mostly retro-/infraauricular area. Dysphagia of some degree was observed in about half of the patients, 3 patients were feeding tube (PEG) dependent prior to RT, 6 patients became PEG-dependent during therapy (dysphagia °III: 5.8%). Radiation-induced side effects (loss of appetite/taste, dysphagia) led to weight loss in 44 patients [2 - 11 kg] due to the complete loss of taste (table 3).

**Table 3:** acute toxicity at completion of treatment and first follow-up

		re-irradiation			macroscopic residual			microscopic residual		
		prior to RT	end of RT	1st f/u	prior to RT	end of RT	1st f/u	prior to RT	end of RT	1st f/u
**mucositis**	I		4			14	0		16	1
	II		1			21	1		18	0
	III		0			5	0		4	0
**dermatitis**	I		2			33	1		27	0
	II		2			10	0		11	0
	III		0			1	0			0
**epitheliolyses**	yes		0			8	0		12	
**xerostomia**	I		3			19	19		21	22
	II					1			0	0
**dysphagia**	I		0	1		11	6		10	5
	II					6	0		4	3
	III					4	0		2	
**weight loss**	yes		1			24	4		19	5
**kg**	median		4			4			5	
	min					2			2	
	max					11			10	
**feeding tube**			0		3	4	4	4	5	
**loss of taste**			2			37			33	
**middle ear effusion**			2	1		11	2		8	2
**otitis**			1			4			6	
**paralysis of facial nerve**		2	2		5	5	5	10	10	9
**ptosis**		3	2		3	1	1			
**reduced jaw opening**		2	2		8	8	3	12	12	5
**xerophthalmia**					1	1		1	1	1
**conjunctivitis**			1	0		6	0		2	0
**lymph edema**							2			2

### Follow-up

Acute toxicity rapidly resolved in most patients, at first follow-up, 12 patients (11.7%) still complained of some difficulty swallowing (°I), 3 (2.9%) reported changes in their diet (°II) while one patient was still dependent on the feeding tube due to dental problems.

Xerostomia °I was reported by 41 patients (39.8%) with symptoms gradually resolving. No skin reactions apart from one case erythema °I and hyperpigmentation °I (1 pt: 1.0%) could be observed (table 3). One patient receiving re-irradiation developed an asymptomatic cystic necrosis of the intracranial tumor part. He is under close clinical and diagnostic follow-up.

#### Outcome

To date, only one patient receiving adjuvant radiation for R1 resection for ACC developed an in-field recurrence. This patient rapidly developed distant metastases (bone, lungs, liver) at the same time and is now undergoing chemotherapy.

Overall best response rate (CR and PR) in patients undergoing re-RT or RT for macroscopic/residual disease is 78.3% (47/60 patients, SD in 15/60 pts). To date, there were 8 complete remissions, one already at the first follow-up (table 4). In the patient group with macroscopic tumor (incl. re-irradiation), three patients developed an in-field recurrence after initial partial response (2 patients receiving re-irradiation, 1 patient receiving primary RT). Both of these patients were re-treated only 19 and 16 months with 51 GyE and 36.2 GyE for acinic cell and mucoepidermoid carcinoma. One patient developed an out-of-field recurrence following re-irradiation, three patients had distant disease progressions.

**Table 4:** initial treatment response

	re-RT	primary/R2
	(N = 16)	(N = 60)
**CR**		8
**PR**	9	30
**SD**	4	11
dna	1	0
pending	2	9
lost to f/u	0	2
		
CR: complete response		
PR: partial response		
SD: stable disease		
dna: does not apply		

**Figure 1:**
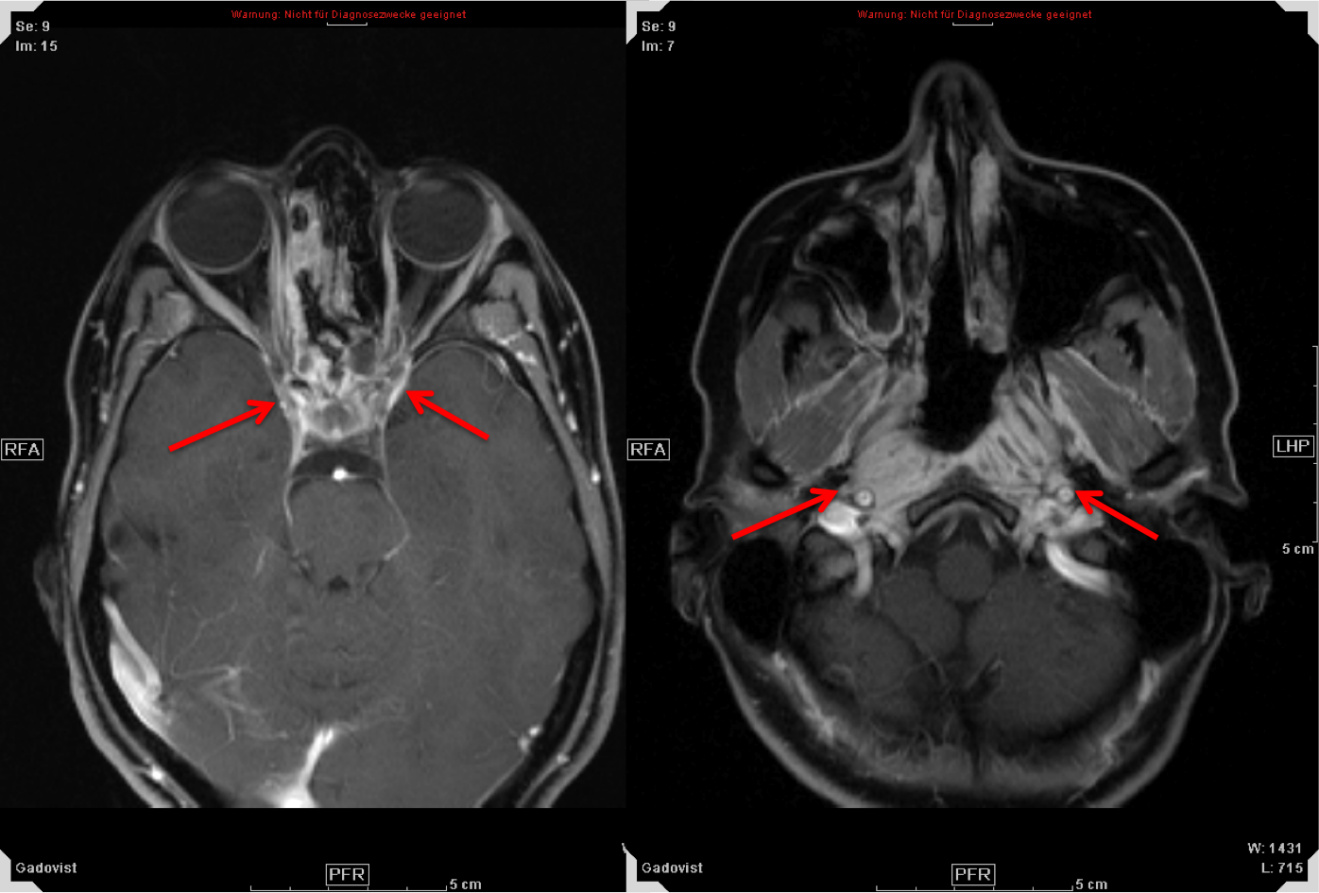
**extensive adenoid cystic carcinoma in 27 year-old lady prior to RT**.

**Figure 2:**
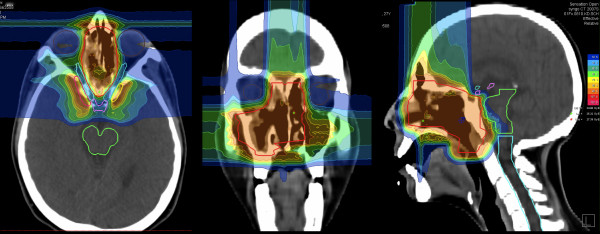
**corresponding carbon ion dose distribution by a 3-field IMPT: 100% corresponding to 24 GyE**.

**Figure 3:**
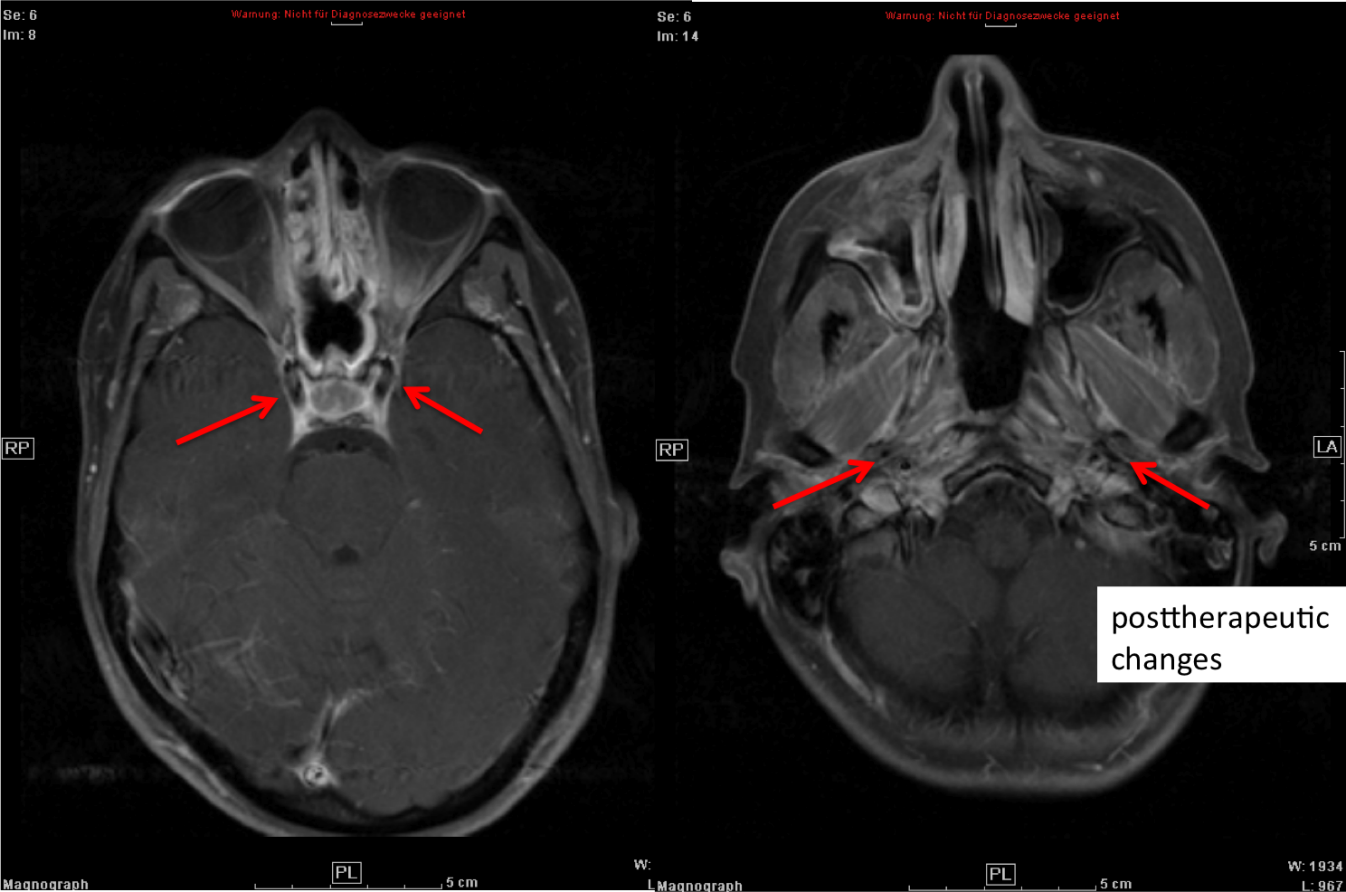
**complete remission 6 months post combined RT (transversal contrast-enhanced MRI)**.

Figures 1, 2, and 3 show a 27 year-old lady with large, partially-resected ACC (CTV1: 238.6 ml, CTV2: 572.2 ml) before (Figure 1) and 6 months after radiotherapy (Figure 3) together with the corresponding carbon ion dose distribution created by a 3-field IMPT (Figure 2).

## Discussion

Treatment was generally tolerated well and without unexpected acute toxicity. Transient alteration of taste, mild xerostomia, and dysphagia were most frequently reported. There was no grade IV or V acute toxicity. These findings are supported by other series using particle radiation [9,11,14].

Despite the sometimes large treatment volumes, we have seen only 6 patients with °III dysphagia (5.8%). Mucositis rates are higher than in our initial or the Japanese series, however, these mostly included tumors located at the base of skull [9,15] and smaller target volumes [11]. Both cohorts [9,11] also did not include R1-resected tumors. In view of treated sites in our patient cohort, occurrence of °III mucositis at roughly 10% is still very low especially considering the fact that the former parotid area was included in 14 out of 40 pts. Also, toxicity does not seem to be increased in the patients without macroscopic residues.

As especially ACCs tend to show perineural infiltration, an independent predictor of local control [4-6], therefore potentially involved neural tracts up to their entrance into the skull base need to be included as proposed by Garden et al [6]. Consequently, this brings higher dose close to the middle ear, therefore, the occurrence of middle ear effusions is not surprising. In all cases, these symptoms completely resolved at first follow-up and are therefore acceptable.

Although it is too early to assess treatment efficacy, response rates and extent of response in the patients with initially macroscopic residues are promising. With our updated results showing no difference in local control between partially resected or primarily irradiated ACCs [16], the role of extensive surgical procedures in the treatment of MSGT hence needs to be reconsidered. With rapidly resolving treatment-related side-effects and very mild late effects [9], the application of extensive and very often mutilating surgical procedures needs to be questioned, especially since inclusion of surgical intervention pathways lead to larger target volumes.

Albeit longer follow-up is needed to assess late effects of re-irradiation with carbon ions, our patients did not experience any major acute radiation-induced toxicity. Observed side-effects were mild, with very fast responses (i.e. resolution of ocular ptosis) occurring even under therapy. It has been shown in various publications that re-irradiation can lead to long-term local control at least in a subset of patients [20-22]. However this is bought at the price of increased late effects with the second course of irradiation. Local control for the second course of irradiation is challenging and also dose-dependent, therefore particle therapy appears to be a logical treatment choice in re-irradiation delivering high doses to the target while sparing surrounding normal tissues to a higher extent than either FSRT or IMRT are able to. While we could - in a small patient number - show that re-irradiation with carbon ions is possible maintaining a low (acute) toxicity profile, late effects still need to be investigated. Two patients in our re-irradiation cohort developed in-field recurrences following re-irradiation, therefore further dose escalation may be explored in a controlled clinical trial setting.

As we hope long-term results with the slightly increased heavy-ion part in the treatment regimen will lead to further improved control rates, none of the local treatment regimen has yet had an impact on overall survival or distant metastasis-free survival in MSGT [2,6,7,9]. The use of concomitant chemotherapy or immunotherapy in squamous cell carcinoma of the head and neck [23,24] has led to a significant improvement not only in local control but also in overall survival. Radiochemotherapy in treatment of MSGT however, has not evolved beyond the phase II-stage or retrospective analysis of very heterogeneous treatment regimen [25-27] so far.

Hence two open questions still remain: can we increase local control at acceptable rates of side-effects in adenoid cystic carcinoma and other malignant salivary gland tumors by increase of the carbon ion RT part? A prospective controlled trial is currently under way to address this issue [17]. The other question is whether patients with adenoid cystic carcinoma will profit from combined treatment with i.e. new substances such as EGFR-inhibitors, which will potentially not increase treatment-related side effects significantly [23,28] in terms of local and distant control. This issue will also shortly be addressed in a prospective phase-II trial [29].

## Conclusion

Achieving local control in MSGT remains challenging. IMRT with carbon ion boost has led to only mild acute side effects in R2-resected tumors of the skull base, toxicity does not seem to be increased in R1-resected tumors of this series. R2-resected patients showed promising treatment response, follow-up is yet too short though to assess long-term local control and potential late effects. Carbon ion therapy for re-irradiation has been shown to be feasible and without significant associated acute toxicity.

## Competing interests

The authors declare that they have no competing interests.

## Authors' contributions

ADJ, AVN, KKH, and MWM were responsible for treatment concepts and patient care, SE, ME for technical treatment planning and quality control, and KKH, JD and MWM for conceptual design. All authors read and approved the final manuscript.

## References

[B1] Spiro RH Salivary neoplasms: overview of a 35-year experience with 2,807 patients Head Neck Surg 1986 8 3 177 84 10.1002/hed.2890080309 3744850

[B2] Chen AM Granchi PJ Garcia J Bucci MK Fu KK Eisele DW Local-regional recurrence after surgery without postoperative irradiation for carciomas of the major salivary glands: implications for adjuvant therapy Int J Radiat Oncol Biol Phys 2007 67 982 987 10.1016/j.ijrobp.2006.10.043 17241753

[B3] Gurney TA Eisele DW Weinberg V Shin E Lee N Adenoid cystic carcinoma of the major salivary glands treated with surgery and radiation Laryngoscope 2005 115 7 1278 82 10.1097/01.MLG.0000165381.64157.AD 15995521

[B4] Mendenhall WM Morris CG Amdur RJ Werning JW Hinerman RW Villaret DB Radiotherapy alone or combined with surgery for adenoid cystic carcinoma of the head and neck Head Neck 2004 26 2 154 62 10.1002/hed.10380 14762884

[B5] Chen AM Bucci MK Weinberg V Garcia J Quivey JM Schechter NR Phillips TL Fu KK Eisele DW Adenoid cystic carcinoma of the head and neck treated by surgery with or without postoperative radiation therapy: prognostic features of recurrence Int J Radiat Oncol Biol Phys 2006 66 1 152 9 10.1016/j.ijrobp.2006.04.014 16904520

[B6] Garden AS Weber RS Ang KK Morrison WH Matre J Peters LJ Postoperative radiation therapy for malignant tumors of minor salivary glands. Outcome and patterns of failure Cancer 1994 73 10 2563 9 10.1002/1097-0142(19940515)73:10<2563::aid-cncr2820731018>3.0.co;2-x 8174054

[B7] Terhaard CHJ Lubsen H Van der Tweel I Hilgers FJM Eijkenboom WMH Marres HAM Tjho-Heslinga RE de Jong JM Roodenburg JL Dutch Head and Neck Oncology Cooperative Group Salivary gland carcinoma: independent prognostic factors for locoregional control, distant metastases, and overall survival: results of the Dutch head and neck oncology cooperative group Head Neck 2004 26 681 92 discussion 692-3 10.1002/hed.10400 15287035

[B8] Terhaard CHJ Lubsen H Rasch CRN Levendag PC Kaanders HHAM Tjho-Heslinga RE van Den Ende PL Burlage F Dutch Head and Neck Oncology Cooperative Group The role of radiotherapy in the treatment of malignant salivary gland tumors Int J Radiat Oncol Biol Phys 2005 61 103 111 10.1016/j.ijrobp.2004.03.018 15629600

[B9] Schulz-Ertner D Nikoghosyan A Didinger B Munter M Jakel O Karger CP Debus J Therapy strategies for locally advanced adenoid cystic carcinomas using modern radiation therapy techniques Cancer 2005 104 2 338 44 10.1002/cncr.21158 15937907

[B10] Douglas JG Koh WJ Austin-Seymour M Laramore GE Treatment of salivary gland neoplasms with fast neutron radiotherapy Arch Otolaryngol Head Neck Surg 2003 129 9 944 8 10.1001/archotol.129.9.944 12975266

[B11] Mizoe JE Tsujii H Kamada T Matuoka Y Tsuji H Osaka Y Hasegawa A Yamamoto N Ebihara S Konno A Organizing Committee for the Working Group for Head-And-Neck Cancer Dose escalation study of carbon ion radiotherapy for locally advanced head-and-neck cancer Int J Radiat Oncol Biol Phys 2004 60 2 358 64 10.1016/j.ijrobp.2004.02.067 15380567

[B12] Huber PE Debus J Latz D Zierhut D Bischof M Wannenmacher M Engenhart-Cabillic R Radiotherapy for advanced adenoid cystic carcinoma: neutrons, photons or mixed beam? Radiother Oncol 2001 59 2 161 7 10.1016/s0167-8140(00)00273-5 11325445

[B13] Münter MW Schulz-Ertner D Hof H Nikoghosyan A Jensen A Nill S Huber P Debus J Inverse planned stereotactic intensity modulated radiotherapy (IMRT) in the treatment of incompletely and completely resected adenoid cystic carcinomas of the head and neck: initial clinical results and toxicity of treatment Radiat Oncol 2006 1 17 10.1186/1748-717X-1-17 PMC1550720 16756669

[B14] Pommier P Liebsch NJ Deschler DG Lin DT McIntyre JF Barker FG Adams J Am Lopes VV Varvares M Loeffler JS Chan AW Proton beam radiation therapy for skull base adenoid cystic carcinoma Arch Otolaryngol Head Neck Surg 2006 132 11 1242 9 10.1001/archotol.132.11.1242 17116822

[B15] Schulz-Ertner D Nikoghosyan A Jäkel O Haberer T Kraft G Scholz M Wannenmacher M Debus J Feasibility and toxicity of combined photon and carbon ion radiotherapy for locally advanced adenoid cystic carcinomas Int J Radiat Oncol Biol Phys 2003 56 391 398 10.1016/s0360-3016(02)04511-x 12738314

[B16] Münter M Umathum V Jensen AD Nikoghosyan A Hof H Jaekel O Debus J Combination of intensity modulated radiation therapy (IMRT) and a heavy ion (C12) boost for subtotal resected or inoperable adenoid cystic carcinomas (ACCs) of the head and neck region J Clin Oncol 2010 28 e16010

[B17] Jensen AD Nikoghosyan A Windemuth-Kieselbach C Debus J Münter MW Combined treatment of malignant salivary gland tumours with intensity-modulated radiation therapy (IMRT) and carbon ions: COSMIC BMC Cancer 2010 10 546 10.1186/1471-2407-10-546 PMC2958954 20937120

[B18] Haberer T Becher W Schardt D Kraft G Magnetic scanning system for heavy ion therapy Nucl Instr Meth Phys Res 1993 330 296 305

[B19] Therasse P Arbuck SG Eisenhauer EA Wanders J Kaplan RA Rubinstein L Verweij J Van Glabbeke M van Oosterom AT Christian MC Gwyther SG New guidelines to evaluate the response to treatment in solid tumors J Natl Cancer Inst 2000 92 205 216 10.1093/jnci/92.3.205 10655437

[B20] De Crevoisier R Bourhis J Domenge C Wibault P Koscielny S Lusinchi A Mamelle G Janot F Julieron M Leridant AM Marandas P Armand JP Schwaab G Luboinski B Eschwege F Full-dose reirradiation for unresectable head and neck carcinoma: experience at the Gustave-Roussy Institute in a series of 169 patients J Clin Oncol 1998 16 3556 3562 10.1200/JCO.1998.16.11.3556 9817275

[B21] Roh KW Jang JJ Kim MS Sun DI Kim BS Jung SL Kang JH Yoo SC Jang HS Chung SM Kim YS Fractionated stereotactic radiotherapy as reirradiation for locally recurrent head and neck cancer Int J Radiat Oncol Biol Phys 2009 74 1348 1355 10.1016/j.ijrobp.2008.10.013 19117695

[B22] Lee N Chan K Bekelman JE Zhung J Mechalakos J Narayana A Wolden S Venkatraman ES Pfister D Kraus D Shah J Zelefsky MJ Salvage re-irradiation for recurrent head and neck cancer Int J Radiat Oncol Biol Phys 2007 68 731 740 10.1016/j.ijrobp.2006.12.055 17379449

[B23] Bonner JA Harari PM Giralt J Cohen RB Jones CU Sur RK Raben D Baselga J Spencer SA Zhu J Youssoufian H Rowinsky EK Ang KK Radiotherapy plus cetuximab for locoregionally advanced head and neck cancer: 5-year survival data from a phase 3 randomised trial, and relation between cetuximab-induced rash and survival Lancet Oncol 2010 11 21 28 10.1016/S1470-2045(09)70311-0 19897418

[B24] Pignon JP le Maitre A Maillard E Bourhis J MACH-NC Collaborative Group Meta-analysis of chemotherapy in head and neck cancer (MACH-NC): an update Int J Radiat Oncol Biol Phys 2007 69 112 114 10.1016/j.ijrobp.2007.04.088 17848275

[B25] Haddad RI Posner MR Busse PM Norris CM Goguen LA Wirth LJ Blinder R Krane JF Tishler RB Chemoradiotherapy for adenoid cystic carcinoma: preliminary results of an organ sparing approach Am J Clin Oncol 2006 29 153 157 10.1097/01.coc.0000203756.36866.17 16601434

[B26] Tanvetyanon T Qin D Padhya T McCaffrey J Zhu W Boulware D DeConti R Trotti A Outcomes of postoperative concurrent chemoradiotherapy for locally advanced major salivary gland carcinoma Arch Otolaryngol Head Neck Surg 2009 135 687 692 10.1001/archoto.2009.70 19620591

[B27] Pederson A Haraf D Blair EA Stenson KM Witt ME Vokes EE Salama JK Chemoreirradiation for recurrent salivary gland malignancies Radiother Oncol 2010 95 308 311 10.1016/j.radonc.2010.03.006 20385414

[B28] Jensen AD Krauss J Weichert W Debus J Münter MW RadioImmunotherapy for adenoid cystic carcinoma: a single-institution series of combined treatment with cetuximab Radiother Oncol 2010 5 102 10.1186/1748-717X-5-102 PMC2987937 21047402

[B29] Jensen AD Nikoghosyan AV Hinke A Debus J Münter MW Combined treatment of adenoid cystic carcinoma with cetuximab and IMRT plus C12 heavy ion boost: ACCEPT [ACC, Erbitux^® ^and particle therapy] BMC Cancer 2011 11 70 10.1186/1471-2407-11-70 PMC3042975 21320355

